# Influence of False Self-Presentation on Mental Health and Deleting Behavior on Instagram: The Mediating Role of Perceived Popularity

**DOI:** 10.3389/fpsyg.2021.660484

**Published:** 2021-04-12

**Authors:** Il Bong Mun, Hun Kim

**Affiliations:** ^1^Department of Media and Communication, SungKyunKwan University, Seoul, South Korea; ^2^Department of Media and Communications, Joongbu University, Goyang-si, South Korea

**Keywords:** lying self-presentation, perceived popularity, depression, deletion, social media

## Abstract

The present study explored motivations (need for approval, impression management) for lying self-presentation on Instagram as well as the mental and behavioral outcomes (depression, perceived popularity, deleting behavior on Instagram) of this presentation. We also examined the differential mediational roles of perceived popularity in accounting for the association between lying self-presentation and depression. Our results showed that individuals with a strong need for approval reported higher levels of lying self-presentation. The results also revealed that lying self-presentation positively influenced depression, perceived popularity and deleting behaviors. Furthermore, we found that even if lying self-presentation increased depression, perceived popularity served as a psychological buffer against depression.

## Introduction

In online environments, people use lying as a way to present themselves. They usually lie to appeal to others regarding physical attraction, age, background and interests (Utz, 2005). In the case of the SNS (Social network service) environment, people have been known to lie about age, gender, job, and relationships status (Wright et al., 2018).

SNSs can accelerate lying self-presentation because users have control over the activities with which they present themselves (Kim and Tussyadiah, 2013). Individuals have relatively no difficulty lying on SNSs, which are characterized by availability, ease of use and anonymity (Kim et al., 2009). Also, in the online environment, people are less likely to detect non-verbal cues related to lies, unlike in the real world (Stanton et al., 2016). The technical tools of social networking services support individuals in creating deceiving self-presentational elements, such as picking and editing images of themselves (Gibbs et al., 2006).

One previous research study found that significant numbers of users believed that their Facebook self was different from their real self, and they exaggerated their positive aspects while minimizing their faults (Gil-Or et al., 2015). Another research study examined false self-presentation on Facebook and classified it into categories of false self-deception, self-comparison and self-exploration. The study confirmed that false self-exploration was the most frequent type of false self-presentation (Michikyan et al., 2015). Given the fact that activities involving visual self-expression, such as photographs or sharing short films, commonly occur on Instagram, it will be necessary to look at the motivations and outcomes of these ways of expressing oneself with lying.

Humans have a basic desire to be approved of by others or groups, an intrinsic desire to be recognized for their value and ability (Rudolph et al., 2005), and this is an important motive to influence individuals' behavior (Homans, 1974). One of these behaviors is self-presentation, which people engage in to gain recognition from others (Hewitt et al., 2003). Particularly, one way to obtain approval is to express one's self deceitfully (McLeod and Genereux, 2008). Lying behavior, like self-expression, is caused by the motivation to win others' approval (Snyder, 1987). Indeed, people either act with selective honesty in order to meet their need for approval, or they properly distort and express themselves by lying (Skinstad, 2008). People with a high level of need for approval paint themselves in a positive light (Schneider and Turkat, 1975), and regardless of their beliefs, either agree with others' values or present themselves with a particular emphasis on similarities (McLeod and Genereux, 2008).

Impression management is not only about controlling and manipulating information about oneself disclosed to others (Schneider, 1981), but also the process of managing one's own impressions of what others perceive (Leary and Kowalski, 1990). An important part of the nature of self-promotion is that it sometimes includes lying in order to sway individuals to agree with one's opinion, which is different from others', in order to win others' goodwill (Feldman et al., 2002). In addition, individuals sometimes can select information about an image strategically and then positively describe their own image (Toma et al., 2008). Although lying behavior for impression management causes moral issues or a confusion in crucial choices (Kupfer, 1982), picking images carefully and editing oneself to display a favorable impression to others have been regarded as universal and essential elements for social interaction (Goffman, 1959). One previous study predicted that respondents who had a high level of impression on others were more likely to lie in their self-presentation (Kashy and DePaulo, 1996). In a study on dating, it was found that people are likely to engage in lying behaviors to appear competent or desirable when first meeting a likable partner (Feldman et al., 2002). Also, in online dating environments, lying behaviors to partners convince individuals that they are getting into a more positive situation than they actually are (Hancock, 2007). Hence, lying is a representative strategy of impression management, and it is a meaningful resource for building an attractive self-presentation.

Popularity acts as a central factor in SNSs (Utz et al., 2012). SNSs also provide an environment or opportunities to produce exaggerated and fabricated information that enables users to easily gain popularity (Zywica and Danowski, 2008). In order to increase popularity on SNSs, some users even purchase SNS accounts to inflate their number of followers (Lagerspetz et al., 2014). To sum it up, lying self-presentation is motivated by gaining popularity from others,41 and lying leads to describing oneself more positively than reality (Hancock, 2007).

Psychological risks have the possibility of affecting deletion behavior. Lying has been regarded as a serious moral violation for many years because it infringes upon the recipient's right to information and freedom of choice (Kupfer, 1982). The lying distributor may suffer from psychological risks such as regret or apologetic feelings due to moral violations, and they may conduct countersteps such as deleting posts or comments to overcome these risks on SNSs (Wang et al., 2011). Individuals are likely to decide whether to delete posts or comments by considering the risks and benefits. Concretely, when uploading a post that is psychologically uncomfortable to another person, owners may recognize the risk and then delete it (Wang et al., 2011). In sum, individuals are likely to be aware of the psychological crisis of both oneself and others that comes with lying behavior, and these risks may soon affect deletion behavior.

Lying may be associated with indicators of emotional adjustment, such as depression, stress, and loneliness (Engels et al., 2006). Likewise, it was suggested that true self-expression reduces depression by reducing emotional labor on Facebook (Grieve and Watkinson, 2016). This result indirectly implies that lying self-presentation requires more emotional labor, which can have a significant effect on depression. Facebook research also identified the effects of lying behavior, which is positively related to psychological factors such as anxiety (Wright et al., 2018). Thus, lying self-presentation is expected to have an impact on depression.

Meanwhile, perceived popularity is likely to affect mental health. Individuals may spend considerable energy in the condition worrying about receiving a negative evaluation of themselves, and people who need to receive support from others may be likely to experience anxiety or depression (Wu and Wei, 2008). In fact, popularity plays a role in predicting loneliness, which is a factor in mental health (Nangle et al., 2003). Research regarding adolescents on social media found that when teenagers perceive a lower level of popularity, they are likely to experience a higher level of depression (Nesi and Prinstein, 2015).

Given these discussions and literatures, the present study is to investigate, in the Instagram environment, how “need for approval” (H1) and “impression management” (H2) affect lying self-presentation, how lying self-presentation affects depression (H3) as well as perceived popularity (H4) and deletion (H5), and the mediating effect of perceived popularity between lying self-presentation and depression (H6).

## Methods

### Participants

Data in this study were collected through an online survey using a quota sampling method to represent in the sample targeting Instagram users. Since the Korea Internet & Security Agency (2019) revealed that Instagram was the second most popular platform in 2019, and Instagram used rate was highest among young adults aged 20–39 years in Korea, the target participants of this study were Instagram users between the ages of 20 and 39 years. The participants were recruited from the EMBRAIN (www.embrain.com) online pool in Korea, a leading online survey company in Korea managing national samples of Korean Internet users. The company maintains over one million internet users whose demographics are similar with those of Korean Internet users. The online survey was conducted from September 18 to October 5, 2019. A total of 1,045 were selected for this study and sent an email with the survey link. We excluded 703 participants who did not meet eligibility criteria or did not complete this survey. The final sample included 315 participants (about 30.1% response rate). About half of them were female (50.2%, *n* = 158), and the mean age of the participants was 29.44 (SD = 5.40). When asked about the amount of time spent on Instagram per day, 40.0% of the participants reported “1–30 min,” 36.7% reported “30 min−1 h,” 15.0% reported “1–2 h,” and 8.3% reported “2 h or more.” The participants also reported uploading an average of 7.7 pictures, videos or other contents (SD = 9.7, range = 0–90), had an average of 132.1 followers (SD = 227.3, range = 1–3,000 followers) and 32.2 followings (SD = 232.6, range = 1–3,000 followings).

### Measures

Need for Approval on Instagram was modified to specifically reflect this study's context from the need for approval questionnaire (Rudolph et al., 2005). In this study, the scale was designed to assess the extent to which participants presented themselves to others in positive terms to obtain the others' approval on Instagram. The subscale consisted of four items which measured on a 7-point Likert scale with anchors of 1 (strongly disagree) and 7 (strongly agree).

Impression Management was developed based on previous studies (Wilson et al., 2014; Keep and Attrill-Smith, 2017). The questionnaire had five questions that probed into a person's attempt to portray him- or herself in a favorable light on Instagram. The items were measured on a 7-point Likert scale with anchors of 1 (strongly disagree) and 7 (strongly agree).

Lying self-presentation was adapted from the Facebook False Self-Presentation Behaviors Inventory (Wright et al., 2018). In this study, LSP was measured using a five-item instrument designed to assess the extent to which participants falsely presented themselves through Instagram. The items were measured on a 7-point Likert scale with anchors of 1 (strongly disagree) and 7 (strongly agree).

Depression was measured with the Depression Scale (Lovibond and Lovibond, 1995), which assesses the symptom severity of depression. In this study, depression consisted of 6 self-report items. Responses were made on a 7-point Likert-type scale, anchored by 1(strongly disagree) and 7 (strongly agree).

Deleting was assessed by a newly created index of two items designed to remove or hide a self-presenting post on Instagram. The items were measured on a 7-point Likert scale with anchors of 1 (strongly disagree) and 7 (strongly agree).

Perceived Popularity was adapted from a previous study (Zywica and Danowski, 2008) that assessed the perception of popularity on SNS. In this study, it was measured using a two-item instrument measured with 7-point Likert scale with anchors of 1 (strongly disagree) and 7 (strongly agree).

The reliability tests of measurements indicated acceptable scores as those with Cronbach's alpha coefficients of more than 0.7 (Table 1).

**Table 1:** Sample items, means, and Cronbach's alpha scores for each construct.

**Constructs**	**Sample items**	**Item means**	**Factor loadings**	**Composite reliability**	**AVE**	**Cronbach's alpha**
Need for Approval	Being liked by users on Instagram makes me feel better about myself.	5.22	0.792	0.88	0.64	0.917
	I feel like a good person when users on Instagram like me.	4.64	0.859			
	When users on Instagram like me, I feel happier about myself.	4.89	0.91			
	I feel proud of myself when users on Instagram like me.	4.60	0.87			
Impression Management	I think my profile is a representation of myself.	5.09	0.788	0.85	0.54	0.770
	I like to create an impact with Instagram posts so that people see me in a certain way.	5.06	0.761			
	I have others' reactions in mind when I post updates to Instagram.	4.67	0.717			
	I'm mindful of how others may perceive me on Instagram.	4.18	0.442			
	I believe that people read a lot about me into the posts that I make on Instagram.	4.68	0.546			
Lying self-presentation	Lying about your relationship status	2.87	0.817	0.90	0.64	0.961
	Lying about your achievements	2.69	0.929			
	Posting or talking about doing something that you didn't actually do on Instagram	2.65	0.928			
	Lying about your hobbies	2.86	0.939			
	Lying about your interests	2.80	0.945			
Depression	I couldn't seem to experience any positive feeling at all.	3.43	0.827	0.89	0.57	0.953
	I found it difficult to work up the initiative to do things.	3.62	0.865			
	I felt that I had nothing to look forward to.	3.43	0.902			
	I was unable to become enthusiastic about anything.	3.55	0.937			
	I felt I wasn't worth much as a person.	3.43	0.883			
Deletion	I often deleted posts that represented myself on Instagram.	3.72	0.848	0.73	0.57	0.871
	I switched posts that represented myself on Instagram so only I could see them (“Save Post”).	3.69	0.914			
Perceived Popularity	Compared to other Instagram users, I am more popular on Instagram.	3.07	0.928	0.85	0.74	0.932
	Other people consider me to be very popular on Instagram.	3.09	0.94			

### Statistical Analyses

All statistical analyses were conducted with path analysis using SEM in Amos 20. To test for the mediating role of popularity in the link between lying self-presentation and depression, we used bootstrapping method (Cheung and Lau, 2008) and the Sobel test was applied (Sobel, 1982). In line with recommendation by Preacher and Hayes (2008), this study generated 5,000 bootstrap samples to estimate a 95% confidence interval (CI) for the indirect effects.

## Results

We performed a confirmatory factor analysis (CFA) to verify factor structure, as well as factorial validity and reliability. A minimum cut off criterion for item deletion is factor loading below 0.50 (Karatepe et al., 2005) and item loadings above 0.50 (Anderson and Gerbing, 1988), composite reliability (CR) values above 0.70 (Molina et al., 2007), Cronbach's alpha above 0.70 (DeVellis, 2003), and average variances extracted (AVE) above 0.50 (Fornell and Larcker, 1981). Factor loadings, Cronbach's alpha values, composite reliability, and AVE were considered acceptable (Table 1) and all squared correlations were less than the AVE.

The results also indicated the fit indices of the research model. The model fits in both models were considered acceptable (Table 2). H1 and H2 stated that the need for approval (H1) and impression management (H2) would influence lying self-presentation. The results showed that need for approval (β = 0.33, CR = 3.74, *p* < 0.001) positively predicted false self-presentation. However, impression management (β = −0.13, *p* = 0.142) was not significant in predicting lying self-presentation. The results demonstrated that only NFA had a positive direct impact on LSP. Hence, H1 and H2 were partially supported.

**Table 2:** Fit indices of measurement and structural models.

**Fit index**	**Recommended value**	**CFA**	**Hypothesized model analysis**	**Mediation analysis**
χ2 (df)		663.33 (237)^***^2.79	735.59 (246)^***^2.99	622.74 (203)^***^
CFI	≥0.90	0.937	0.928	0.934
IFI	≥0.90	0.938	0.928	0.934
TLI	≥0.90	0.927	0.919	0.925
RMSEA	≤ 0.08	0.076	0.079	0.079
PCLOSE	≤ 0.05	0.000	0.000	0.000
PGFI	≥0.50	0.669	0.683	0.675

*** *p < 0.001*.

**Figure 1:**
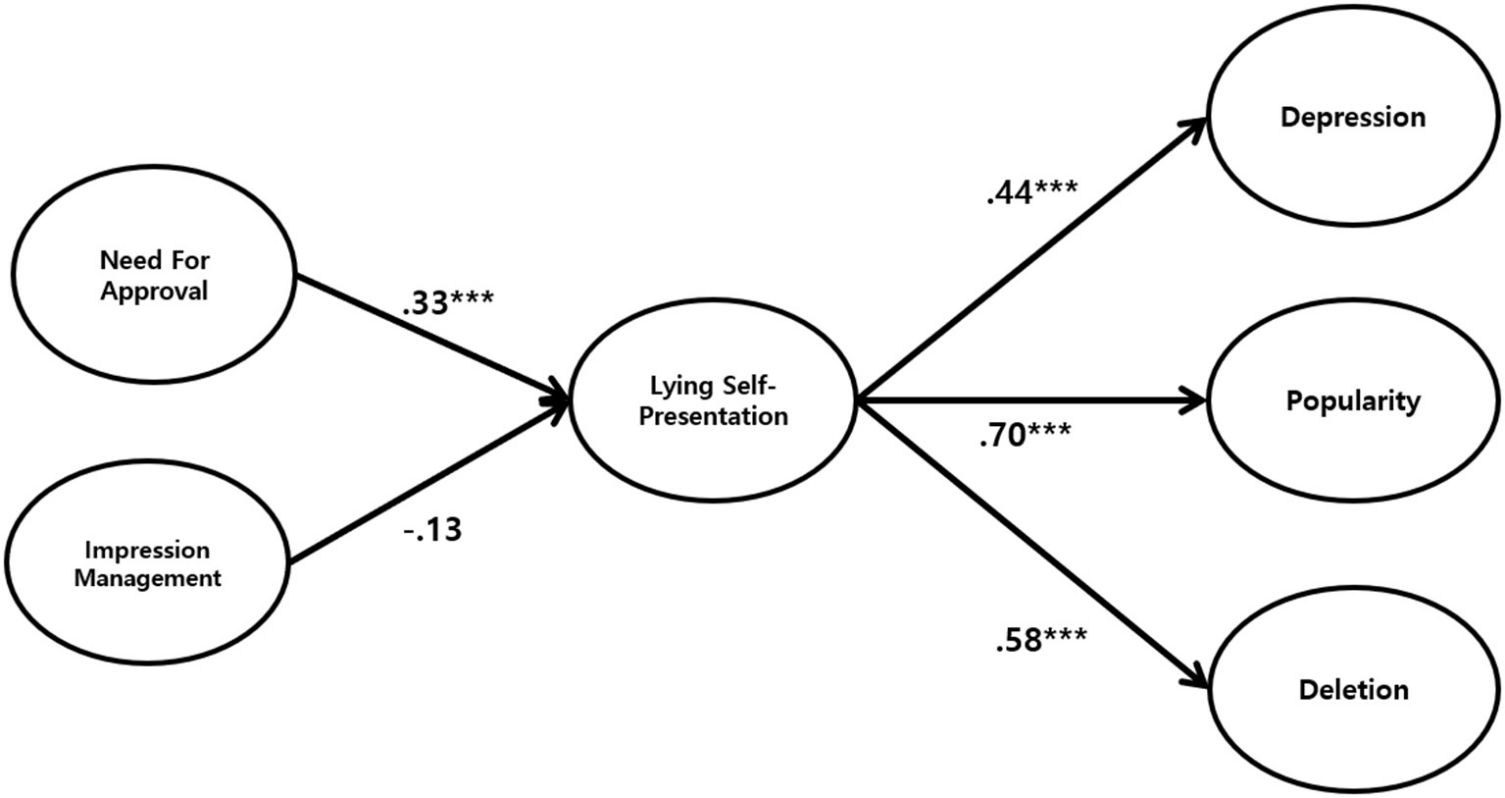
SEM results of the hypothesized path model. Path values are unstandardized coefficients. ***p < 0.001.

H3, H4, and H5 stated that lying self-presentation would influence depression (H3), perceived popularity (H4), and deletion of posts (H5). As expected, the significance testing results showed that lying self-presentation had a positive effect on depression (β = 0.44, CR = 7.81, *p* < 0.001), perceived popularity (β = 0.70, CR = 14.02, *p* < 0.001), and deletion of posts (β = 0.58, CR = 9.48, *p* < 0.001), respectively (Figure 1). Thus, H3, H4, and H5 were supported.

In the mediation analyses, the SEM was revealed to be an acceptable fit for the data (Table 3). H6 stated that perceived popularity would mediate the relationships between lying self-presentation and depression. As shown in Table 3, the direct effect was 0.60 (CR = 7.40, *p* < 0.001), and the indirect effect was −0.16 (*p* < 0.01). The Sobel test indicated that the mediated effect was significant (z = −2.53, *SE* = 0.006, *p* < 0.01). Thus, when lying self-presentation predicted depression, popularity partially mediated the significance of both the direct and indirect effects. Lying self-presentation had a significant effect on depression and decreased when perceived popularity was added as the mediating factor.

**Table 3:** Bootstrap analyses of the magnitude and statistical significance of indirect effects.

**Model pathways**	**Total Effect**		**Direct effect**		**Indirect effect**	
	**β(SE)**	**95% CI**	**β(SE)**	**95% CI**	**β(SE)**	**95% CI**
LSP → PO → Dp						
LSP → Dp	0.44 (0.05)^**^	0.54 to 34	0.60 (0.08)^**^	0.75 to 0.46	−0.16 (0.06)^**^	−0.06 to −0.29
LSP → PO	0.70 (0.04)^**^	0.77 to 0.62	0.70 (0.04)^**^	0.77 to 0.62	–	–
PO → Dp	−0.23 (0.09)^**^	−0.08 to −0.40	−0.23 (0.09)^**^	−0.08 to −0.40	–	–

*LSP, False self-presentation; PO, Perceived Popularity; Dp, Depression. These values are based on unstandardized path coefficients*.

** *p < 0.01*.

## Discussion

This study aimed to examine (a) psychological predictors of lying self-presentation, (b) the influence of lying self-presentation on psychological and behavioral outcomes on Instagram and (c) the mediating effects of perceived popularity.

The results showed that need for approval had an important role to play in engaging behaviors related to lying self-presentation. These results show that self-presentation is a principal means of acquiring approval. The results also identified that lying self-presentation might be a way of being approved by other users on Instagram. This finding is inconsistent with previous findings that people attempt to engage in selective honesty to meet their need for recognition (Skinstad, 2008) and that self-popularity positively affects lying behaviors. People with a high approval motivation tend to use social media improperly (Takao et al., 2009), and this tendency has also been confirmed in the self-presentation context. Considering that the need for approval positively affects emotional well-being, those with a need for approval may acquire psychological well-being and be less conscious of others' negative perception due to lying behaviors.

In contrast, the relationship between impression management and lying self-presentation was not significant. Unlike previous results that say that lying behavior is one of the important strategies for impression management (Hancock, 2007), the relationship was not supported in SNS situations. This might be caused by the environmental factors of Instagram. The rate of communicating with strangers on Instagram is 58%, which is higher than Facebook (38%), while the probability of communicating with acquaintances on Instagram is only 22% (Yang and Lee, 2020). Instagram users may perceive lying to strangers as a higher risk behavior. In fact, SNS users may engage in lying behavior involving impression management in order to establish a social relationship (Underwood et al., 2011), but they may be less interested in establishing impression management on Instagram with strangers because they perceive the risk involved. Also, given the fact that impression management with lying has a negative effect on future relationship goals, it is expected that individuals engage in lying self-presentation for long-term impression management rather than short-term. Previous research has also suggested that the magnitude of lying behavior should be controlled by considering future interactions with others (Toma et al., 2008).

Next, our study showed that lying self-presentation online was positively associated with psychological and behavioral outcomes. Supporting the study's predictions, lying self-presentation significantly increased depression, deleting posts and popularity. On Facebook, honesty-based activities were part of predictors to increase subjective well-being (Kim and Lee, 2011), and also there have been significant correlations found between mental health, such as anxiety, and lying self-presentation behavior (Wright et al., 2018). Similar to these studies, our findings show that lying self-presentation positively affects mental health such as depression.

Third, lying self-presentation had a direct or indirect effect on depression when mediated by popularity. The results imply that even if behaviors of lying self-presentation increase the users' level of depression, the depression of these people can be reduced by popularity. The results suggest that when people engage in behaviors of lying self-presentation, they may become popular on Instagram and accordingly feel decreased levels of depression. These results provide that perceiving oneself in a popularity state may serve as a psychological buffer against negative health outcomes.

Fourth, lying self-presentation was identified as a factor affecting deletion behavior. This study found the meaningful mechanism that lying self-presentation leads to actual behavior related to a SNS as well as psychological outcomes. Psychological risks caused by lying behavior are likely to affect deletion on a SNS. Specifically, the psychological risk related to lying behavior can be divided into risks perceived by oneself and others (Wang et al., 2011). If the false expression is for social interactions, individuals may possibly delete their own content, taking into account the psychological risks to others. Tufekci (2008), for example, suggested that individuals who focus on strong ties in an online environment are less likely to engage in lying acts such as aliasing. Based on this finding, future research could address the level of ties as a predictor between lying self-expression and deletion behavior.

Finally, in a comparison of lying-self presentation (Wright et al., 2018), individuals were more likely to engage in lying behaviors on Instagram (M = 2.77, Likert scale = 7) than Facebook (M = 1.14, Likert scale = 6). In addition, while the relationship between lying self-presentation and depression was not significant on Facebook (Wright et al., 2018), lying self-presentation on Instagram increased depression. Our study showed that lying self-presentation on Instagram might be different from that on Facebook.

The limitations of this study should be noted. It is important to understand why and what functions, such as profile, posting, liking, and comments, are used for lying self-presentation on SNSs because providers can selectively put more technical resources into situations where lying self-presentation stands out. Therefore, it is proposed that future research should check functions' specific effect on lying self-presentation.

## Conclusion

In this study, we explored how false self-presentation was associated with unhealthy online communication behaviors such as deleting self-presenting posts on Instagram as well as with negative mental health attributes. For future studies, this research provides a greater understanding of the effects of false self-presentation on actual use behavior in the SNS context. Also, our findings expand the available database regarding psychosocial correlates of false self-presentation in that lying behavior may negatively impact mental outcome but can also reduce negative mental health when mediating perceived popularity. Future research should consider all the positive and negative aspects of self-presentation on social media. The most meaningful finding of this study is that popularity can buffer the relationship between false self-presentation and depression. In particular, the relevance of perceived popularity and buffering effects in online environments is meaningful because it expands the scope of research from that of previous studies, which confirmed only the buffering effects of social support (Cummins, 1990) and religiosity (Wills et al., 2003).

## Data Availability Statement

The raw data supporting the conclusions of this article will be made available by the authors, without undue reservation.

## Ethics Statement

Ethical review and approval was not required for the study on human participants in accordance with the local legislation and institutional requirements. The patients/participants provided their written informed consent to participate in this study.

## Author Contributions

IM and HK designed the research, conducted the literature searches, and wrote the manuscript. IM collected the research data and performed statistical analysis. HK reviewed and revised the final manuscript. All authors contributed to the article and approved the submitted version.

## Conflict of Interest

The authors declare that the research was conducted in the absence of any commercial or financial relationships that could be construed as a potential conflict of interest.
